# Price Analysis of Over‐the‐Counter Retinol Products

**DOI:** 10.1111/jocd.70361

**Published:** 2025-07-17

**Authors:** Aarushi K. Parikh, Jules B. Lipoff

**Affiliations:** ^1^ Robert Wood Johnson Medical School Rutgers University New Brunswick New Jersey USA; ^2^ Department of Dermatology, Lewis Katz School of Medicine Temple University Philadelphia Pennsylvania USA

**Keywords:** economics, over‐the‐counter, pricing, retinol, skincare

To the editor,

Retinols are known for multifaceted roles in skincare and dermatology. Specifically, retinols are touted for their anti‐aging properties [1], diminishing the appearance of fine lines and wrinkles. In widespread use in over‐the‐counter (OTC) skincare products, the pricing of these products greatly varies. Previously, we observed variations in the prices of OTC dermatology products (OTC minoxidil prices varying by gender) unrelated to the amount of active ingredient in the product [2]. Similarly, we aimed to study what factors may be associated with the prices of various OTC retinol products.

This study was exempt from institutional review board approval. From October 20 to December 21, 2023, data on retinol products were collected from 9 retailers (Bluemercury, CVS, Neiman Marcus, Nordstrom, Sephora, Target, Ulta Beauty, Walgreens, Walmart) in Pennsylvania and New Jersey. Information gathered included brand name, type of skin care product, packaging, manufacturer's suggested retail price, size, unit price of the product, disclosure of retinol percentage, retinol amount and unit price of retinol (if applicable), and labeling notes on other ingredients.

Descriptive statistics and Pearson's coefficient were analyzed using Microsoft Excel and Python. Data collection excluded duplicates, retinol alternatives, products without measurable mL of retinol, and online‐only items. The analysis utilized the retail prices obtained from the initial store where the product was sourced. A paired *t*‐test confirmed consistent pricing across stores for the same product, revealing no statistically significant differences (*p*‐value > 0.05).

We identified 151 unique retinol products across 9 stores; 66 disclosed their retinol percentage (Table 1). No significant price difference was found between products disclosing and not disclosing retinol percentage (*p*‐value > 0.05).

**TABLE 1:** Products disclosing retinol percentage and associated prices.

Type	Number of products disclosing	Median retail price of products (USD)	Median unit price of products (USD/mL)	Median retinol percentage	Median unit price of retinol (USD/mL)
Cleanser	0/4	—	—	—	—
Eye cream	1/6	12.99	0.87	3	28.87
Face oil	6/11	56.65	2.4	0.75	333.33
Moisturizer	5/29	74.00	2.18	0.7	311.69
Scrub	0/1	—	—	—	—
Serum	38/70	52.75	1.82	0.5	301.58
Treatment	17/28	83.00	3.05	1	331.67
Total	66/151	62.00	2.16	0.5	331.67

There was no statistically significant strong correlation observed between the percentage of retinol and neither the retail price nor the unit price of the product (Figure 1). There was an apparent weak negative correlation between the unit price of retinol and the percentage of retinol; as the amount of retinol in the product increased, the price/mL of retinol tended to decrease in a linear fashion.

**FIGURE 1:**
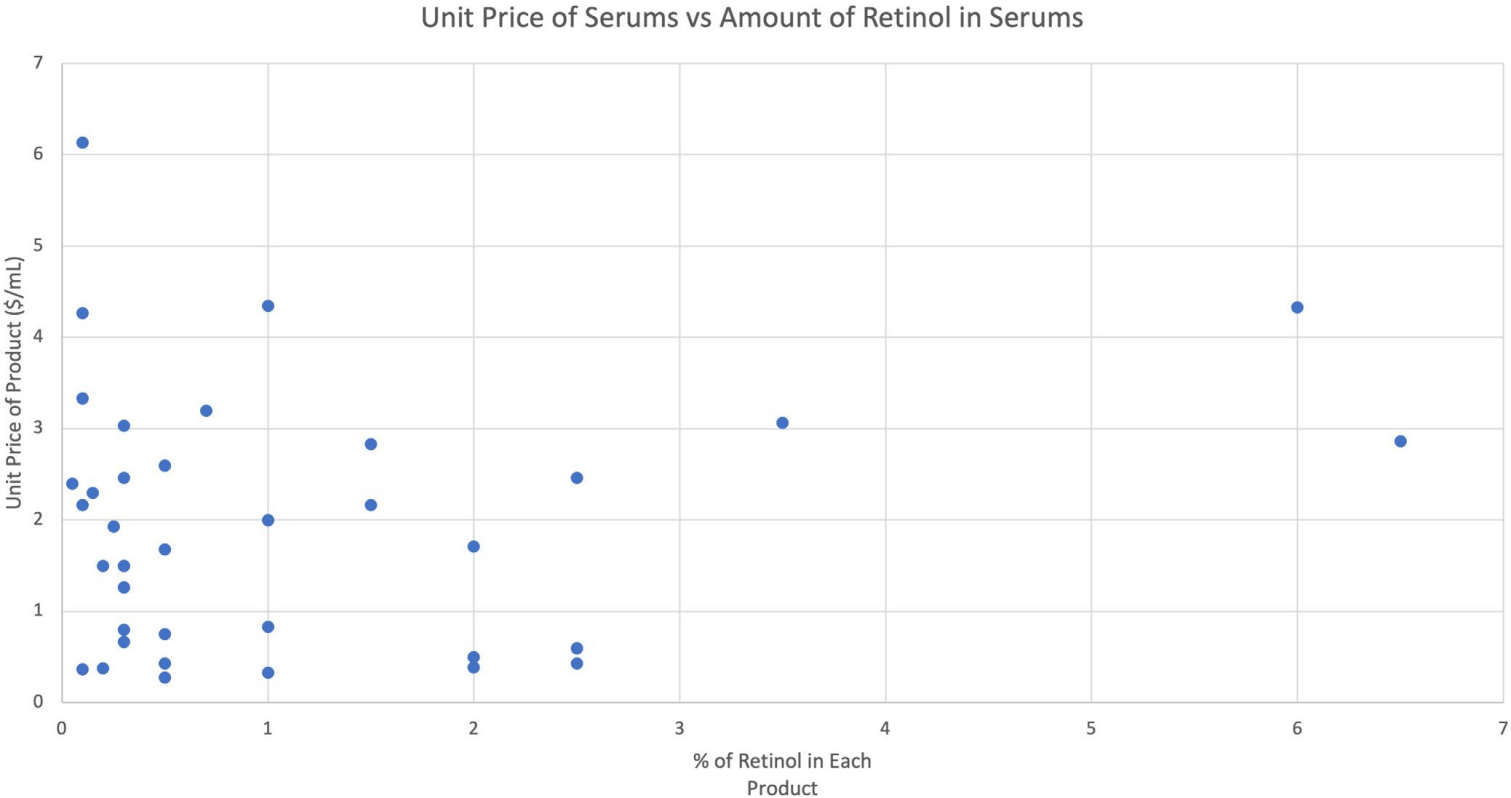
Scatterplot of Unit Price (USD/mL) of Serum Products and Percentage of Retinol in Serum Products.

Additionally, there was no statistically significant difference identified between packaging types or for products marketing themselves with additional so‐called natural ingredients/fragrances when comparing retail unit price, unit price of retinol, and product percentages of retinol.

Our results suggest that the price of retinol OTC products is not strongly related to the amount or concentration of the retinol active ingredient. Instead, the price of OTC retinol products may be influenced by factors such as brand positioning, with evidence of divergent pricing strategies within categories possibly suggesting differences in formulation and delivery systems (such as encapsulated retinol) or reflecting the marketing and perceived credibility. Our study was limited by the analysis products available in these specific stores and in this United States region.

We acknowledge that retinols' efficacy is not determined by concentration; it may be affected by packaging—which protects products from light and air, prevents contamination, and maintains formulation integrity—and higher concentrations may not necessarily translate to greater efficacy, as encapsulated formulations offer gradual release for better stability and reduced irritation, while non‐encapsulated retinol provides a faster release but may cause more sensitivity [3]. With the average American average spending $322.88/year on skincare [4], informed consumers should consider product pricing alongside important factors such as formulation design rather than solely relying on brand claims. Dermatologists may advise patients that a quality retinol product is one suited to their specific needs. Future studies should investigate how other factors beyond retinol content (e.g., retinol stabilization, type of vehicle, formulation design) relate to product prices. In conclusion, our results suggest that prices of retinol products are not correlated with retinol ingredient content, and consumers should be informed about important factors that may contribute to the efficacy of retinol products.

## Author Contributions

All authors concept and design; acquisition, analysis, or interpretation of data; drafting of the manuscript; critical revision of the manuscript for important intellectual content. Aarushi K. Parikh: conceptualization, writing–original draft, writing–revisions, data collection, statistical analysis. Jules B. Lipoff: conceptualization, writing–revisions, supervision.

## Disclosure

Dr. Lipoff reports receiving personal fees from AlphaSights, Amgen, Boehringer Ingelheim, Guidepoint Global, LLC, and RBC Consultants; royalties for a book from Springer. Science & Business Media; and grants from Pfizer outside the submitted work.

## Ethics Statement

The authors have nothing to report.

## Consent

The authors have nothing to report.

## Conflicts of Interest

The authors declare no conflicts of interest.

## Data Availability

The data that support the findings of this study are available from the corresponding author upon reasonable request.

## References

[1] T. Quan , “Human Skin Aging and the Anti‐Aging Properties of Retinol,” Biomolecules 13, no. 11 (2023): 1614. 38002296 10.3390/biom13111614 PMC10669284

[2] M. R. Wehner , K. T. Nead , and J. B. Lipoff , “Association Between Gender and Drug Cost for Over‐the‐Counter Minoxidil,” JAMA Dermatology 153, no. 8 (2017): 825–826. 28593214 10.1001/jamadermatol.2017.1394 PMC5817599

[3] C. W. Shields, 4th , J. P. White , E. G. Osta , et al., “Encapsulation and Controlled Release of Retinol From Silicone Particles for Topical Delivery,” Journal of Controlled Release 278 (2018): 37–48. 29604311 10.1016/j.jconrel.2018.03.023

[4] C. Moore , “Americans Will Spend $15G on Lifetime Skincare, This Cosmetics Retailer Claims.” Fox Business, 2021, https://www.foxbusiness.com/lifestyle/americans‐spend‐15g‐lifetime‐skincare‐cosmetics‐retailer‐claims.

